# Matrix Intensification Affects Body and Physiological Condition of Tropical Forest-Dependent Passerines

**DOI:** 10.1371/journal.pone.0128521

**Published:** 2015-06-24

**Authors:** Justus P. Deikumah, Clive A. McAlpine, Martine Maron

**Affiliations:** The University of Queensland, Landscape Ecology and Conservation Group, School of Geography, Planning and Environmental Management, Brisbane, Qld 4072, Australia; Hungarian Academy of Sciences, HUNGARY

## Abstract

Matrix land-use intensification is a relatively recent and novel landscape change that can have important influences on the biota within adjacent habitat patches. While there are immediate local changes that it brings about, the influences on individual animals occupying adjacent habitats may be less evident initially. High-intensity land use could induce chronic stress in individuals in nearby remnants, leading ultimately to population declines. We investigated how physiological indicators and body condition measures of tropical forest-dependent birds differ between forest adjacent to surface mining sites and that near farmlands at two distances from remnant edge in southwest Ghana. We used mixed effects models of several condition indices including residual body mass and heterophil to lymphocyte (H/L) ratios (an indicator of elevated chronic stress) to explore the effect of matrix intensity on forest-dependent passerines classed as either sedentary area-sensitive habitat specialists or nomadic generalists. Individual birds occupying tropical forest remnants near surface mining sites were in poorer condition, as indicated by lower residual body mass and elevated chronic stress, compared to those in remnants near agricultural lands. The condition of the sedentary forest habitat specialists white-tailed alethe, *Alethe diademata* and western olive sunbird, *Cyanomitra obscura* was most negatively affected by high-intensity surface mining land-use adjacent to remnants, whereas generalist species were not affected. Land use intensification may set in train a new trajectory of faunal relaxation beyond that expected based on habitat loss alone. Patterns of individual condition may be useful in identifying habitats where species population declines may occur before faunal relaxation has concluded.

## Introduction

Matrix intensification, the replacement of lower-contrast matrices with high-contrast ones such as surface mining, is an increasingly common phenomenon in many tropical landscapes. It can directly and/or indirectly lead to habitat loss, fragmentation, pollution and loss of farmlands [1–3]. Loss of native vegetation through clearing and extraction of timber resources near and within existing forest remnants can also be facilitated by the easier access to forest that can result from matrix intensification [4].

Intensification of the landscape matrix can increase fragmentation impacts on wildlife communities within adjacent remnants [5,6], through reducing habitat resources, increasing edge effects, altering disturbance regimes, modifying microclimates, and increasing invasion and human pressures [5,7]. Surface mining, in particular, creates an inhospitable matrix that can impede dispersal through the landscape [8]. Such matrix intensification could therefore present several environmental stressors, potentially resulting in negative long-term consequences for individuals, and potentially the local population to which they belong [9].

While there are immediate local changes that matrix intensification brings about, the influences on individuals occupying adjacent habitats may be less evident initially. This is because during time-lagged ecological responses to habitat disturbances, populations tend to initially crowd into remaining intact patches [10] but decline rapidly over the succeeding trajectory of faunal relaxation [11]. Thus, short-term increases in population densities within remnant habitats immediately following the disturbance of surrounding landscapes may provide a biased impression of the conditions within remnants [12].

Growing empirical evidence suggests that the health and condition of individuals can be used to reveal relationships between animals and their environments that are not evident from species occurrence and distribution models [13–15]. Anthropogenically-mediated chronic stress can lead to population declines and ultimate extinction [16]. Organisms experiencing chronic stress may experience immune system impairment, muscle wasting, and reduced growth and reproduction rates [16–18]. Thus, early identification of spatial patterns of chronic stress can help identify potential threats (including poor quality habitats) to populations before they start to decline. For example, indicators of body condition and stress have been used to indirectly quantify habitat quality and suitability [19,20]. Stress metrics have also been used to identify mechanisms behind population changes and to examine effects of fluctuations in environmental resources on populations [13].

In birds, indicators of condition can often be easily measured and reliably linked to individual survival and fitness [21]. Two classes of measure that are useful are body condition and physiological stress. Body condition includes any measure of the accumulated energy in the body, such as body mass or fat scores, and provides an indication of the health and condition of that individual [22–24]. Physiological condition and stress indices relate to the physiological and behavioural strategies adopted by individual animals that can result in the overstimulation of coping mechanisms (short-term stress) in response to prolonged exposure to environmental stressors [25,26]. An indicator of persistent exposure to stressors is the ratio of heterophils to lymphocytes in peripheral blood, as corticosterone (the main stress hormone in birds) increases heterophil levels while depressing production of lymphocytes [27]. Elevated chronic stress can indicate individuals in poor physiological condition with lower fitness [20,28–31].

Despite the strong relationship between indicators of body and physiological condition and factors such as survivorship [32–36] and breeding success [19,37,38] of individuals, relatively few studies have explored spatial variation in indices of individual condition [13,14,39–41]. Most of these studies have either compared individuals in fragmented vs. contiguous forests [20] or in smaller patches vs. larger ones [39]. It has been suggested that understanding spatial patterns of individual condition may reveal initial responses to recent landscape changes that may in turn translate to population-level changes, as faunal relaxation proceeds [42]. If influences on individuals can be detected before populations start to decline, then appropriate conservation priorities can be set more efficiently before the full impact of landscape change is realised [42].

In this study, we investigated how physiological indicators and body condition measures of tropical forest-dependent birds differ in native remnants adjacent to surface mining sites and those near farmlands, at two distances from remnant edge (near/far). We used mixed effects models of several condition indices including body condition and heterophil to lymphocyte (H/L) ratio (an indicator of chronic or long-term stress) to explore the effect of landscape change and matrix intensification on two contrasting categories of tropical forest-dependent passerines: sedentary area-sensitive habitat specialists vs. nomadic highly mobile generalists. We hypothesised that the sedentary habitat specialists inhabiting forest adjacent to more-intensive mining matrices will be in poorer condition than those inhabiting forest near agricultural land, but that this pattern would be less evident for the highly-mobile habitat generalists.

## Methods

### Study area

The study was conducted in the fragmented upper Guinea forest landscape of south-west Ghana (3° 5`W-1° 10`E; 4° 35`N-11°N). The forest areas of Ghana are confined to the Guinea-Congolian zone, and are highly fragmented as a result of clear-fell logging for high-value timber products and rapid human population growth [43]. Cleared areas are used for raising cash crops and food crops, and are exposed to frequent fires [43]. This has led to the fragmentation of a formerly contiguous forest into distinct patches within a non-forest matrix. The area is also rich in minerals such as gold, bauxite, and iron ore, and their extraction is a serious threat to the region’s forests [43]. Gold mining is often located adjacent to, and within, forest reserves and many large-scale surface gold mining operations have recently been established in the region [2,44,45].

Like in many developing countries, the mining industry in Ghana has expanded over the past 30 years due to change in economic policies [45]. The rapid expansion of the mining industry, exacerbated by poor livelihoods in Ghana’s tropical forest areas, has led to the increase in small-scale mining in and near forest patches. The forest fragments of south-west Ghana are surrounded by a land use matrix dominated by cropland, which consists of small farms and fallow land. Relictual native forest trees are scattered within these croplands, and cacao farms in particular usually retain a canopy of native emergent tree species [43,46]. However, forests once adjacent to a ‘softer’ matrix of low-intensity farmland increasingly now abut the highly inhospitable matrix of surface mining [47].

### Ethics statement

All field work and experimental protocols were approved by the University of Queensland Animal Ethics Committee under permit number GPEM/191/10. Permission to access conservation reserves was granted by the Forestry Commission and the Wildlife Division of Ghana. Permission to access private company properties (e.g. mining sites adjacent to reserves) was granted by relevant authorities [Please contact Mr Kwaku Sefah (Human Resources Manager and Mr William Addo (Environment and Safety Manager), AngloGold Ashanti (Iduapriem) Limited/Reg. No. 303018 for future permissions].

### Case-study species

Four passerine species were selected for comparison based on pre-existing knowledge of their habitat preference and the fact that they are still widespread in the study area [48,49]. Two are sedentary habitat specialists that are mostly associated with primary and secondary tropical rainforests, although they occasionally venture into open and edge habitats [50]. These are the white-tailed alethe, *Alethe diademata* and western olive sunbird, *Cyanomitra obscura*. *A*. *diademata* is a ground-foraging insectivore from the family Turdidae, and is restricted to the upper Guinean forest zone [51] with some evidence of population decline in parts of its range [52]. *C*. *obscura* is one of the most insectivorous sunbirds [53,54].

The other two target species were the yellow-whiskered greenbul, *Andropadus latirostris* and little greenbul, *Andropadus virens*. Both are habitat generalists, often associated with secondary forests and are mainly nomadic [55]. *A*. *virens* prefers second-growth and edge habitats, and feeds mostly on insects while *A*. *latirostris* inhabits all types of primary and secondary forest exploring both interior and edges including degraded forest including regenerating young second growth [55]. They are omnivorous, feeding on fruits and a wide variety of invertebrates and occasionally a few vertebrates [55].

### Experimental design

The study was conducted in the two main forest types of south-west Ghana: evergreen and semi-deciduous forests. Forty study sites were selected in 20 forest patches (2–579 km^2^ in area) in forest reserves, national parks and sacred groves (areas protected by local taboo or as royal burial grounds); the forest edge nearest to each site was adjacent to either surface mining or agriculture. Each patch was located > 2 km from other forest patches. In each patch, two paired study sites (interior and edge) were located. Edge sites were located within 50 m from the forest boundary and the interior sites at least 500 m away from the boundary. Each site consisted of a 500 m-long line transect. Transects were placed such that each was internally homogeneous as far as possible with respect to canopy closure and density of large trees.

#### Morphological and haematological measurements

At each site, birds of the focal species were targeted for capture in mist nets in both the dry (November, 2010 to April, 2011) and rainy (May-September, 2011) seasons. Five-shelved nets with 15 x 15 mm mesh, 2.7–3.2 m high, were used. During each visit to a study site, three mist nets were placed at 100–150 m intervals along each transect and were kept open (closed during rain and high wind) from 0600 hr -1700 hr and inspected at regular intervals of 20–30 minutes. All species captured were identified.

After capture, morphological measurements were taken from each bird including total mass, bill length, bill width, wing length, visible subcutaneous fat, brood patch score; pectoral muscle shape, tail length and tarsus length following the methods outlined in Pyle [56] and the ringer’s manual of the British Trust for Ornithology [57]. The average of two measurements of each morphological attribute was used in all analyses. Sex could not be determined with confidence for most of our case study species because these species are not sexually dimorphic except for *C*. *obscura* [56]. Subcutaneous fat deposits were quantified according to scales developed by Helms et al. [58].

Peripheral blood samples (100–150 μl) were taken from captured birds within 2–3 minutes of capture using brachial venipuncture. A thin coat of blood smear was made on individually marked microscope slides, air-dried, fixed in absolute ethanol and stained with Wright-Giemsa solution.

Blood smears were examined and the proportions of different leucocytes (lymphocytes, heterophils, basophils, monocytes, and eosinophils) were determined at x400 magnification. Examination was halted when 100 leucocytes had been found following the procedure of Verso [59]. Thrombocytes were excluded because they normally present irregular, aggregated distributions that might reduce accuracy [60]. The counts of leucocytes were repeated (at least twice) and the average count was used for analyses [39]. Variability in blood smear quality can bias leucocyte differential counts [39]. To account for this the number of smudged cells (likely both red and white blood cells) in 10 frames was counted and averaged to obtain a smudge index that was used in the analysis [39].

Data gathered from morphological attributes was used to derive residual body mass used as an indicator of body condition while those from differential leukocyte counts yielded information on H/L ratio used as an indicator of elevated chronic stress of each case-study bird species.

#### Explanatory variables

The total area of each forest patch was calculated by digitization from 1:50,000 Google Earth Maps. The boundaries of forest patches were easily visually distinguished from surrounding crop lands, plantations, and surface mining areas [43] using ArcGIS 10 [61]. From Google Earth Maps, we characterized vegetation in three classes, namely: forest, cropland, and abandoned farmlands. We calculated the total area of forest habitat within 1 km and 5 km buffer distances from each study location.

Vegetation surveys were conducted to characterise the structure and composition of the vegetation at each site. Tree species with diameter at breast height (dbh) > 60 cm (large trees) were counted in five randomly placed 20 x 20 m quadrats at each site. Within the same 20 x 20 m quadrats we counted all fruiting and flowering plants. Within five randomly selected 5 x 5 m quadrats we visually estimated ground cover, including grass, litter and bare ground at each site. Where appropriate, all measured or count data were converted to values per hectare or per square metre. The logging history of each forest patch was collated from literature and recorded [43,62].

Microclimatic conditions at each sample station were also measured as these conditions may influence habitat selection and bird behaviour [63–65]. We measured air temperature (°C), relative humidity (%) and wind speed (m/s) using Kestrel 3000 wind meter/anemometer weather station (K3000 Nielsen-Kellerman) on three occasions and averaged the values for each sample station.

#### Data exploration

Prior to all analyses, data were tested for normality using Shapiro-Wilk tests, and for homogeneity of variance using Levene's test [66] and log-transformed where appropriate. All statistical analyses were performed in R [67]. All explanatory variables were standardized to have a mean of zero and standard deviation of 1. We tested for collinearity among the local-scale explanatory variables using Spearman’s correlation coefficient. Pairs of explanatory variable with high correlation can be considered as proxies of one another [68,69]. For pairs of explanatory variables that had coefficients of correlation > | 0.5|, the explanatory variable with least influence on any response variable was removed from the final analyses. Micro-climate measures, shrub density and flowering trees correlated with density of large trees and were therefore not used in the final analysis (S1 Table).

#### Statistical modelling

Principal component analysis (PCA) was computed from a correlation matrix using all morphological measurements [70]. Principal component analysis (PCA) is often used to develop indices of the overall body size or condition in avifauna based on morphometric measurements [70]. The first principal component (PC1) from this analysis was used as an index of structural size [18]. Residual body mass was determined from a linear regression of absolute body mass using PC1 as a predictor of body mass [18]. Relationships between residual body mass and direct body condition measures (subcutaneous fat and pectoral muscle scores) were assessed using Spearman’s rank correlation test. Most of the condition indicators measured can fluctuate with season and time of day. Pearson correlation tests were used to examine the relationship between time since sunrise, Julian date and season of the year on all condition indices for each case study species (S3 Table).

Generalized linear mixed effects models (GLMMs) with model averaging based on AICc were used to assess variation in body and physiological condition indices of each case-study species between matrix types and distance from edge categories [71]. The effects of both landscape and local-scale vegetation variables were ranked based on their importance in influencing body and physiological parameters of each species. GLMMs were fitted with Gaussian family error structure and implemented using lme4 [72]. Five predictor variables were involved in the modelling of each response variable, resulting in a total of 64 models. Models were ranked according to summed AICc weights and parameter values were averaged across models within four delta (∆) values of the best model for each response using the MuMln package in R [73]. Model-averaged coefficients estimated for all six explanatory variables and their effect sizes were used to explain the differences in body condition (residual body mass), chronic stress (H/L ratio) and subcutaneous fat deposit of habitat specialist and generalist species.

## Results

Residual body mass was normally distributed (Shapiro-Wilk normality test, p > 0.05) with homogenous variance (Levene’s test, p>0.05); H/L ratio and subcutaneous fat score were not, and so were log-transformed before all analyses. A total of 126 and 105 individuals of *Andropadus latirotris* and *Andropadus virens* (both habitat generalists) and 116 individuals of *C*. *obscura* respectively were trapped in 38 of the 40 sites while 59 individuals of *Alethe diademata* were trapped in 36 sites. Mean (± SD) values of all measurements of all condition of each case study species are summarised in S2 Table. A summary of Pearson correlations between condition indices of each case study species with time since sunrise and Julian date are presented in S3 Table. Scatterplots of these data were also examined to check for potentially nonlinear relationships, but none were identified.

Five predictor variables were used in the final modelling. These include matrix types, distance to remnant edge, density of large trees, density of fruiting trees and forest extent. The three vegetation covariates varied between matrix types and distance to remnant edge categories (see S1 Fig). Density of large trees was higher in agricultural sites and in interior habitats compared to mining sites and near remnant edges. Mean number of fruiting trees was also higher in sites near agricultural matrices compared to surface mining (S1 Fig).

**Fig 1:**
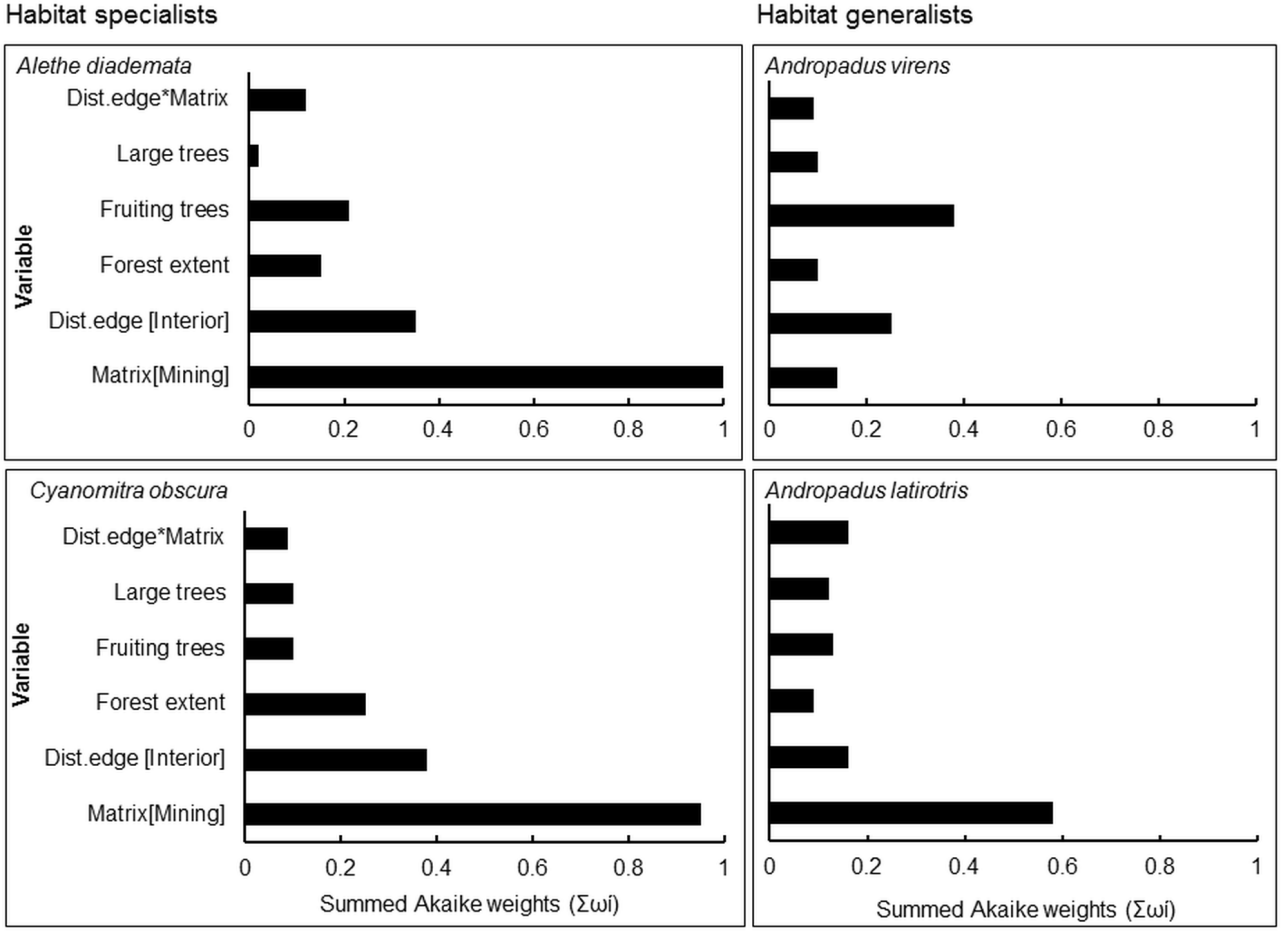
Summed Akaike weights (Σω_i_) from model averaging of each explanatory variable and their relative importance in influencing body condition as indicated by residual body mass of both habitat specialists and generalists.

**Fig 2:**
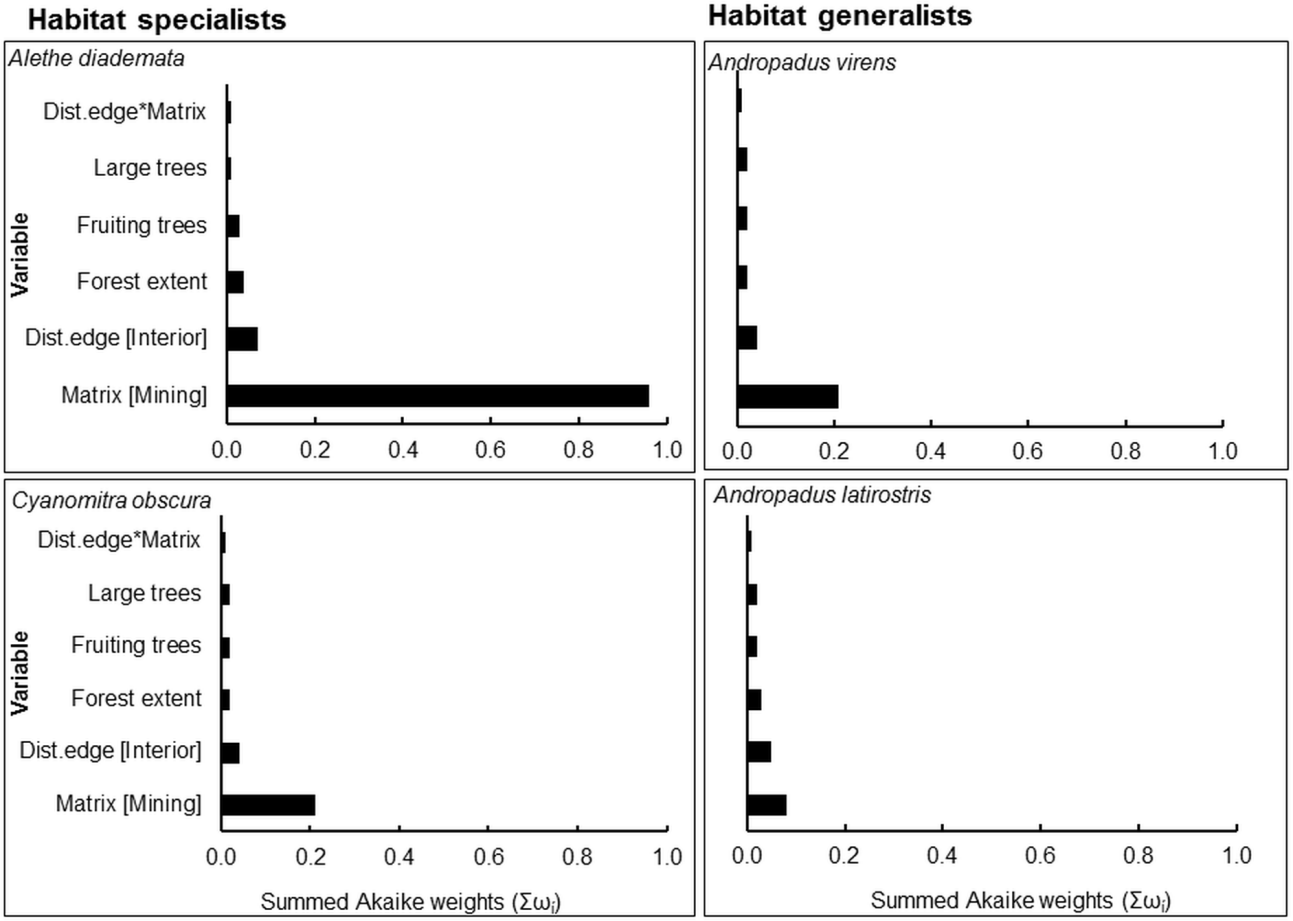
Summed Akaike weights (Σω_i_) from model averaging of each explanatory variable and their relative importance in influencing elevated chronic stress as indicated by H/L ratio of both habitat specialists and generalists.

**Fig 3:**
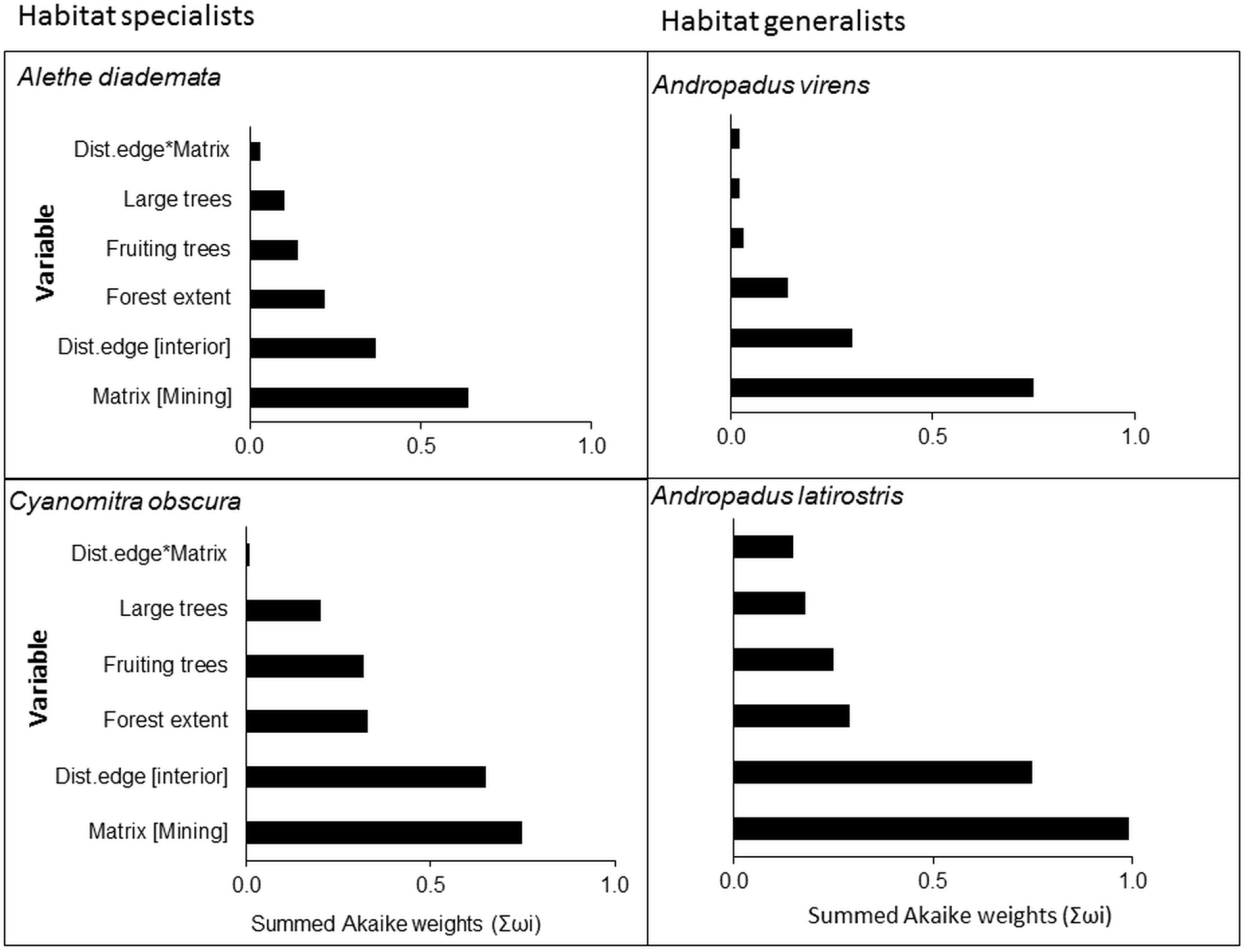
Summed Akaike weights (Σωi) from model averaging of each explanatory variable and their relative importance in influencing elevated chronic stress as indicated by subcutaneous fat scores of both habitat specialists and generalists.

**Fig 4:**
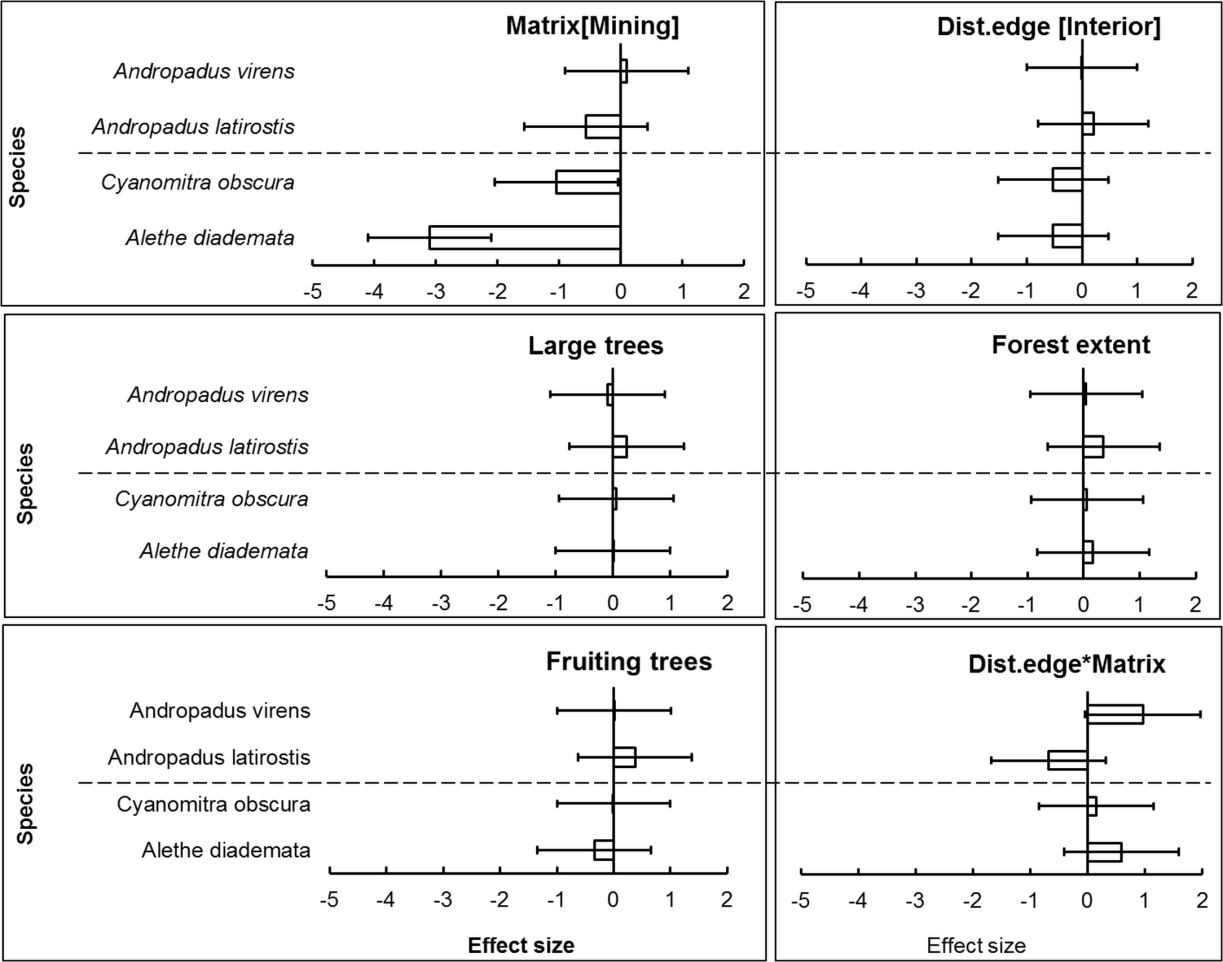
Model-averaged coefficients (error bar = 95% CI) of explanatory variables’ influence on residual body mass of all case study species. Generalist above the horizontal dashed line and specialists below in each panel.

**Fig 5:**
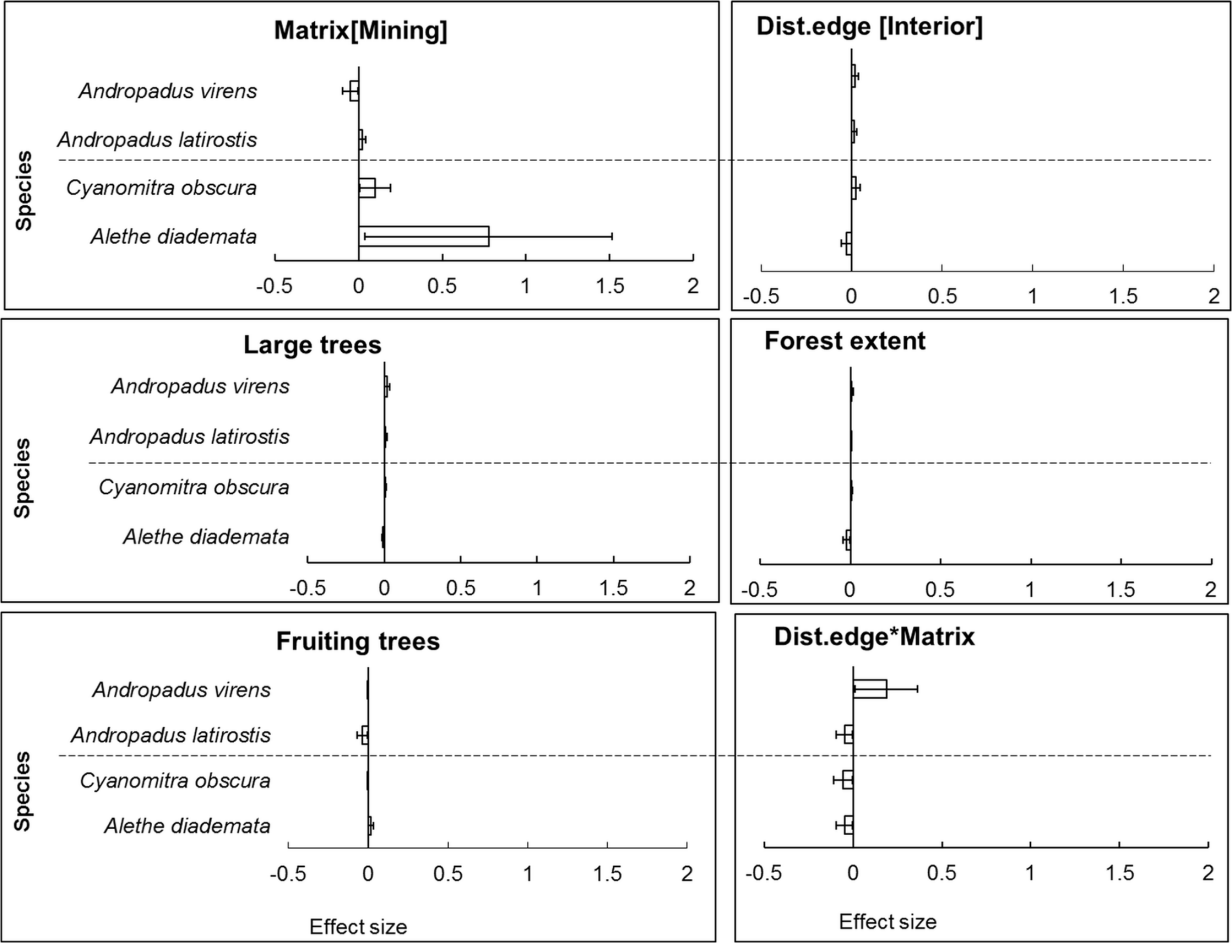
Model-averaged coefficients (error bar = 95% CI) of explanatory variables’ influence on H/L ratio of all case study species. Generalist above the horizontal dashed line and specialists below in each panel.

Model-averaged results revealed that adjacency to a mining matrix was the most important influence on indicators of body condition and elevated chronic stress levels of habitat specialists, *A*. *diademata* and *C*. *obscura*. Matrix type had the highest rank importance, as indicated by the Akaike weights summed across models in the 95% confidence set (Σω_i_), for residual mass of *A*. *diademata* and *C*. *obscura*, and H/L ratio of *A*. *diademata* (Figs 1 and 2). Neither distance from edge nor any of the key vegetation covariates were useful predictors of body (as indicated by residual mass) and physiological condition (H/L ratio) of any species. Distance to patch edge had a positive influence on subcutaneous fat score for *A*. *diademata*, but the summed Akaike weight for this variable was relatively low (Σω_i_ = 0.64), while mining matrix ranked highest (Σω_i_ = 0.75) and negatively influenced fat scores of *C*. *obscura* (Fig 3) (see also S4 Table). The 95% confidence intervals of all averaged model parameters included zero except for the matrix effects on residual body mass and H/L ratio of the specialists *A*. *diademata* and *C*. *obscura* (Figs 4 and 5).

**Fig 6:**
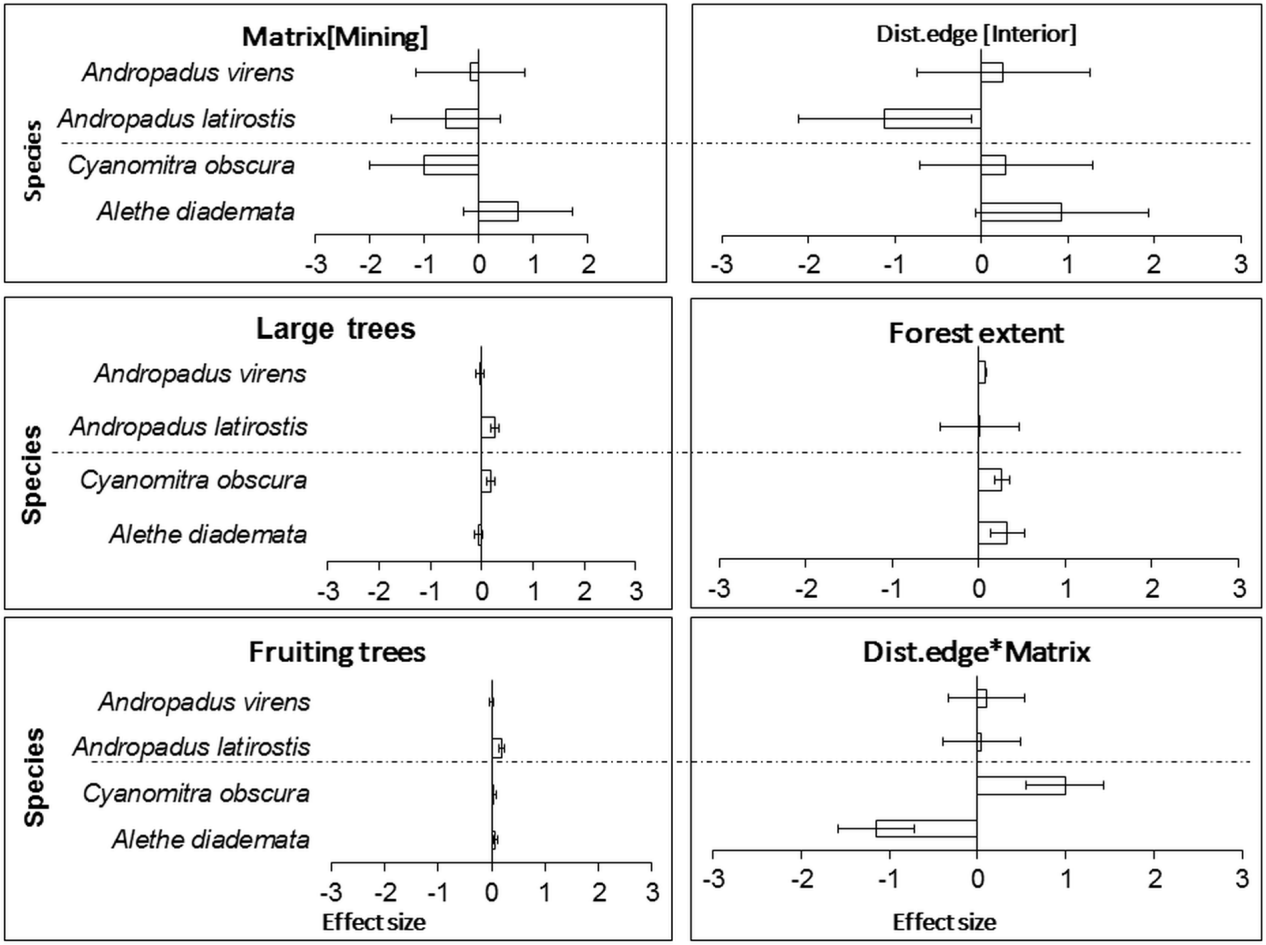
Model-averaged coefficients (error bar = 95% CI) of explanatory variables’ influence on subcutaneous fat score of all case study species. Generalist above the horizontal dashed line and specialists below in each panel.

As predicted for habitat generalists, neither matrix type nor proximity to remnant edge significantly influenced the residual mass (Fig 1), fat score or H/L ratio (Fig 2) of *A*. *latirostris* or *A*. *virens* (Figs 3 and 6) (see also S4 Table).

## Discussion

Our results indicate that matrix intensification can negatively affect body and physiological condition of birds occupying adjacent native remnants. We found that habitat specialists occupying remnants near surface mining sites were in poorer condition, as indicated by lower residual mass and higher H/L ratio (suggesting elevated chronic stress), compared to those in remnants near agricultural lands. Our results suggest that increasing matrix intensity adjacent to native remnants could reduce the fitness of individuals occupying those remnants, ultimately leading to local population declines.

### Matrix effects on condition indices

Matrix type had an important influence on the two species of habitat specialists as indicated by lower residual mass and elevated chronic stress of birds trapped in remnants near surface mining sites. This indicates that birds living in remnants near high-intensity land use matrices may be in poorer body and physiological condition than their conspecifics in remnants near agricultural sites. Individuals with lower residual mass may have lower energy reserves [74] and may be less able to endure starvation [75]. A higher proportion of heterophils in circulating blood is a useful indication of elevated chronic stress in birds [76–80]. Elevated H/L ratio has been associated with numerous environmental stressors [14,15,76].

Long-term elevated stress levels can have deleterious effects on growth rate and cause severe protein loss, and can ultimately reduce fecundity and survival of individuals [80,81]. These effects on individual fitness may ultimately lead to population-level consequences [35]. For example, during the 1998 El Nino event, a severe reduction in algal food sources triggered elevated corticosterone levels of individual Galapagos marine iguanas (*Amblyrhynchus cristatus*) which resulted in reduced survivorship of the affected population [35]. Also, reduced availability of lipid-rich fish food triggered by climate shifts in the mid-1970s resulted in elevated levels of corticosterone and decreased growth in nestlings of the red-legged kittiwakes (*Rissa brevirostris*) [34]. This affected post-fledging survival and recruitment, resulting in population decline [34].

Sex and age can both influence body condition and blood parameters [18,82,83]. However, in the current study, we could sex only one of our case study species, and so sex was not included in the final models. It is therefore conceivable that the patterns we observed were confounded by local differences in sex ratios or age structure related to matrix land use. Such influences of patch characteristics on age structure and sex ratio have been observed for other species [84–86].

Direct interpretation of body mass indices is not straightforward [22,23,87]. Thus, in this study, multiple factors such as fat and muscle scores were measured as alternative indicators of body condition. Poor body condition has been linked to elevated chronic stress associated with food shortage [19,35,39,88]. Vegetation clearing during mining may have directly resulted in rapid changes in availability of habitat resources such as fruit and arthropod biomass in the matrix itself. Even the forest specialists we studied, *A*. *diademata* and *C*. *obscura*, are known occasionally to venture into and access resources within adjacent farmland when the habitat structure is suitable [55,89]. The loss of these complementary resources might reduce the foraging efficiency of individuals of sedentary species whose home ranges previously overlapped with the affected area. Lack of food may compel individual birds to direct their energy storage in the form of subcutaneous fat into maintenance that may have led to reduced body mass [90].

Conversion of heterogeneous farmland to surface mining may also indirectly affect habitat quality in nearby remnants through secondary anthropogenic influences. Following conversion to surface mining in our study area, remnants can become more accessible to local inhabitants whose livelihood originally depended heavily on farming [91]. From these forest patches local people remove fuel wood, non-forest timber products (NFTPs), building materials and medicinal plants [92,93]. Some farmers also now engage in illegal mining and lumbering in and around remnants within surface mining landscapes because their farmlands have been converted to mines [1,91]. These activities may have resulted in removal of fruiting and flowering trees and loss of vegetation structure, and this may affect resource availability for birds within these remnants. This observation was supported by variations detected in all three vegetation covariates (density of large trees, fruiting trees and extent of forest) between matrix type and distance from patch edge (S1 Fig).

Another pathway through which mining might affect birds in adjacent remnants is contamination by heavy metals. Several cases of mercury, arsenic and cyanide contaminations have been reported in plants [94,95], drinking water [96], fish [97], and human serum [98] in the study area. It is possible that birds may eat invertebrates and fruits that may have traces of these chemicals. This could contribute to the differences in patterns in elevated chronic stress reported in this study in surface mining sites, and therefore requires further investigation.

### Variation in response to matrix intensification: habitat specialists vs. generalists

We predicted that while the conversion of low-intensity agriculture to high-intensity surface mining is likely to influence condition of sedentary habitat specialists, such patterns should be less pronounced in mobile generalists. We caution that because the central hypothesis of this study was tested with only four case study species (N = 4) we cannot generalise about the responses of other forest specialists and generalists. Nonetheless, our results were consistent with this prediction, with *Alethe diademata* and *C*. *obscura* (sedentary specialists) most negatively affected by adjacent surface mining. These differences are likely to be due to the different way in which sedentary specialists and wide-ranging generalists use the landscape.

Sedentary habitat specialists tend to be more strongly affected by fragmentation impacts than are habitat generalists [12,99]. Habitat specialists may require specific food resources, be less mobile and are often restricted to interior habitats [7]. Terrestrial forest insectivores such as those we studied are among the least-mobile avifaunal groups [100]. Thus, movement across the inhospitable surface mining matrices may be challenging for this group, potentially increasing the stress associated with accessing other nearby remnants. This can increase their sensitivity to landscape modification compared to highly mobile generalists [7,101]. Although habitat generalists may also face challenges of inter-patch movement with changes in surrounding matrix land use, they may have greater dispersal ability and be more able to make use of resources in alternative fragments if the original target of movement is hard to access [101]. The omnivorous generalist birds (*Andropadus virens and Andropadus latirostris*) in this study may switch their diet if their primary food source is unavailable [102] allowing them more flexibility in accessing resources. Nevertheless, the generalist *A*. *latirostris* did have somewhat lower fat scores in remnants adjacent mining areas, and so effects may not be limited to more sedentary species.

## Conclusion

The original concept of extinction debt refers to extinctions yet to occur during faunal relaxation after habitat loss [103,104]. However, the carrying capacity of a landscape is likely to be also affected by the hospitability of the matrix [40]. When the matrix is changed to a more intensive land use, it may set in train a new trajectory of faunal relaxation beyond that expected based on habitat loss alone. Patterns of individual condition can be useful in identifying species and populations potentially carrying an extinction debt. If habitats where species may be at high risk of population declines can be identified and hence species experiencing faunal relaxation through measures of condition indices, then pre-emptive conservation measures could be taken to prevent local extinction [39]. Strategic approaches to clearing of farms adjacent to native vegetation and revegetation following mine decommissioning should target the improvement of habitat heterogeneity in the matrix, particularly near remnant edges.

## Supporting Information

S1 Fig Mean (±SD) plots of key site-level vegetation characteristics, (a) density of large trees; (b) extent of forest habitat within 1km^2^ and (c) number of fruiting trees of mist netting between matrix type and proximity to remnant edge. (TIF)

S1 Table Correlation matrix of explanatory variables. Coefficients in **bold** shows highly correlated variables that were excluded in the analyses.
(DOCX)

S2 Table Condition indices of four target species (two habitat generalist and two specialists) captured in sites adjacent to agricultural and mining matrices at two distances to remnant edge. (DOCX)

S3 Table Summary of Pearson correlation coefficients between condition indices of case study species, time of day, date and season of the year. (DOCX)

S4 Table Model-averaged coefficients from models of residual body mass, H/L ration and fat score for each target species and sum of Akaike weights (Σωi) for each explanatory variable. (DOCX)

S5 Table Model data behind all analyses. (XLSX)
