# Supplementary figures and images for: Matrix Intensification Affects Body and Physiological Condition of Tropical Forest-Dependent Passerines
Source: PLoS One. 2015 Jun 24;10(6):e0128521. doi: 10.1371/journal.pone.0128521 (PMC4479600; doi:10.1371/journal.pone.0128521)

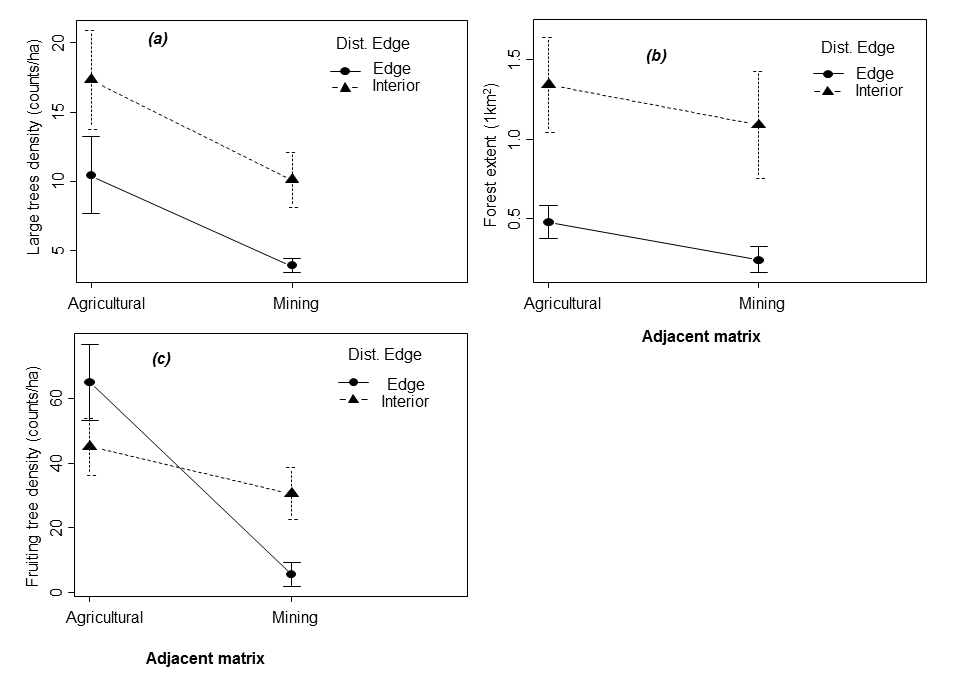

Supplement: S1 Fig — (TIF) [file pone.0128521.s001.tif]
