# Supplementary material for: Matrix Intensification Affects Body and Physiological Condition of Tropical Forest-Dependent Passerines
Source: PLoS One. 2015 Jun 24;10(6):e0128521. doi: 10.1371/journal.pone.0128521 (PMC4479600; doi:10.1371/journal.pone.0128521)
Supplement: S1 Table — Coefficients in bold shows highly correlated variables that were excluded in the analyses. (DOCX) [file pone.0128521.s002.docx]

**S1 Table**: Correlation matrix of explanatory variables. Coefficients in **bold** shows highly correlated variables that were excluded in the analyses

| Explanatory variables | 1 | 2 | 3 | 4 | 5 | 6 | 7 | 8 | 9 | 10 | 11 | 12 |
| --- | --- | --- | --- | --- | --- | --- | --- | --- | --- | --- | --- | --- |
| 1. Dist. Edge | 1 |  |  |  |  |  |  |  |  |  |  |  |
| 2. Matrix | 0 | 1 |  |  |  |  |  |  |  |  |  |  |
| 3. Canopy cover | **0.7** | -0.1 | 1 |  |  |  |  |  |  |  |  |  |
| 4. Shrub density | **-0.8** | 0.3 | **-0.7** | 1 |  |  |  |  |  |  |  |  |
| 5. Fruiting trees | -0.2 | -0.2 | -0.1 | 0 | 1 |  |  |  |  |  |  |  |
| 6. Flowering trees | -0.2 | -0.2 | -0.1 | 0.1 | **0.7** | 1 |  |  |  |  |  |  |
| 7. Large trees | 0.5 | -0.2 | **0.7** | **-0.6** | 0.1 | 0.1 | 1 |  |  |  |  |  |
| 8. Wind speed | **-0.6** | 0 | -0.5 | 0.5 | 0.1 | 0.1 | -0.5 | 1 |  |  |  |  |
| 9. Temperature | -0.2 | 0.1 | -0.3 | 0.3 | -0.4 | -0.3 | -0.3 | 0.4 | 1 |  |  |  |
| 10. Relative humidity | 0.3 | -0.3 | 0.3 | -0.3 | 0.4 | 0.4 | 0.2 | -0.4 | **-0.7** | 1 |  |  |
| 11. Forest extent (1 km^2^) | **-0.6** | 0.1 | -0.3 | 0.4 | 0.1 | 0 | -0.1 | 0.3 | 0.1 | -0.1 | 1 |  |
| 12. Forest extent (5 km^2^) | **0.6** | -0.1 | 0.4 | -0.4 | -0.1 | 0 | 0.2 | -0.4 | -0.1 | 0.2 | **-1** | 1 |
