# Supplementary material for: Matrix Intensification Affects Body and Physiological Condition of Tropical Forest-Dependent Passerines
Source: PLoS One. 2015 Jun 24;10(6):e0128521. doi: 10.1371/journal.pone.0128521 (PMC4479600; doi:10.1371/journal.pone.0128521)
Supplement: S2 Table — (DOCX) [file pone.0128521.s003.docx]

**S2 Table:** Condition indices of four target species (two habitat generalists and two specialists) captured in sites adjacent to agricultural and mining matrices at two distances to remnant edge.

| Condition indices | Agricultural matrix(mean ± SD) | | Mining matrix (mean ± SD) | |
| --- | --- | --- | --- | --- |
|  | Edge | Interior | Edge | Interior |
| *Andropadus virens* |  |  |  |  |
| Residual mass | -0.06 (0.34) | -0.07 (0.30) | -0.016 (0.28) | 0.13 (0.44) |
| Haematocrit (%) | 0.54 (0.05) | 0.53 (0.06) | 0.51 (0.05) | 0.54 (0.06) |
| Body mass (g) | 26.34 (1.20) | 26.01 (1.54) | 25.29 (0.88) | 24.68 (1.47) |
| Haemoglobin(g/L) | 167.87 (18.79) | 166.87 (10.49) | 159.26 (14.97) | 164.89 (15.08) |
| Lymphocytes (%) | 57.98 (4.97) | 80.18 (55.90) | 80.56 (53.97) | 58.10 (8.07) |
| Heterophils (%) | 14.42 (2.79) | 14.86 (3.01) | 13.55 (3.66) | 14.28 (5.66) |
| H/L ratio | -0.61 (0.07) | -0.69 (0.21) | -0.74 (0.22) | -0.63 (0.18) |
| Subcutaneous fat | 2.58 (1.28) | 3.56 (0.69) | 1.42 (0.92) | 2.64 (1.51) |
| *Andropadus latirostris* |  |  |  |  |
| Residual mass | 0.52(1.75) | 0.61(0.99) | -0.76 (1.20) | -0.25 (2.06) |
| Haematocrit (%) | 0.54 (0.07) | 0.53 (0.02) | 0.52 (0.05) | 0.53 (0.05) |
| Body mass (g) | 28.43(1.80) | 28.89 (1.16) | 25.86 (1.91) | 26.12 (1.46) |
| Haemoglobin (g/L) | 167.76 (16.60) | 182.09 (19.02) | 174.06 (16.45) | 174.3 (11.16) |
| Lymphocytes (%) | 75.99 (3.52) | 75.95 (6.2) | 73.80 (9.26) | 72.38 (6.07) |
| Heterophils (%) | 13.15 (3.14) | 11.26 (2.10) | 13.30 (1.51) | 13.28 (1.52) |
| H/L ratio | 0.17 (0.04) | 0.15 (0.03) | 0.18 (0.02) | 0.19 (0.06) |
| Subcutaneous fat | 2.30 (1.10) | 3.47 (1.09) | 1.68 (1.01) | 2.93(1.26) |

| Condition indices | Agricultural matrix(mean ± SD) | | Mining matrix (mean ± SD) | |
| --- | --- | --- | --- | --- |
|  | Edge | Interior | Edge | Interior |
| *Alethe diademata* |  |  |  |  |
| Residual mass | 1.91 (2.27) | -1.64 (1.35) | 1.28 (1.43) | -1.54 (1.72) |
| Haematocrit (%) | 0.52 (0.05) | 0.51 (0.08) | 0.51 (0.05) | 0.55 (0.07) |
| Body mass (g) | 35.36 (2.23) | 34.99 (1.45) | 31.76 (1.32) | 31.94 (1.72) |
| Haemoglobin(g/L) | 160.21 (19.55) | 174.48 (20.72) | 153.63 (12.15) | 160.94 (17.74) |
| Lymphocytes (%) | 70.04 (13.85) | 70.19 (11.94) | 60.69 (6.44) | 59.47 (10.64) |
| Heterophils (%) | 8.62 (3.69) | 8.15 (4.68) | 14.11 (2.81) | 12.86 (5.640) |
| H/L ratio | 0.12(0.05) | 0.12(0.05) | 0.23 (0.08) | 0.23 (0.12) |
| Subcutaneous fat | 0.67 (0.83) | 2.485 (1.47) | 1.29(1.29) | 1.17(1.17) |
| *Cyanomitra obscura* |  |  |  |  |
| Residual mass | 0.40 (0.97) | 0.62 (0.99) | -0.48(1.04) | -1.09(1.37) |
| Haematocrit (%) | 0.58 (0.06) | 0.57 (0.06) | 0.56 (0.06) | 0.56 (0.07) |
| Body mass (g) | 10.18 (0.39) | 10.24 (0.39) | 10.12 (0.34) | 10.34 (0.40) |
| Haemoglobin(g/L) | 180.40 (18.35) | 187.85 (26.89) | 163.52 (21.27) | 174.90 (26.25) |
| Lymphocytes (%) | 12.93(4.31) | 13.44 (4.71) | 13.49 (3.25) | 14.40 (3.21) |
| Heterophils (%) | 70.73 (8.85) | 66.43 (8.15) | 58.45 (7.76) | 62.65 (5.81) |
| H/L ratio | 0.18 (0.05) | 0.21 (0.08) | 0.23 (0.04) | 0.23 (0.06) |
| Subcutaneous fat | 2.19 (1.03) | 2.70 (1.37) | 1.12 (1.11) | 2.37 (0.99) |

**S2** **Table** (continued)
