# Supplementary material for: Matrix Intensification Affects Body and Physiological Condition of Tropical Forest-Dependent Passerines
Source: PLoS One. 2015 Jun 24;10(6):e0128521. doi: 10.1371/journal.pone.0128521 (PMC4479600; doi:10.1371/journal.pone.0128521)
Supplement: S3 Table — (DOCX) [file pone.0128521.s004.docx]

**S3 Table:** Summary of Pearson correlation coefficients between condition indices of case study species, time of day, date and season of the year

| Condition indices | Julian date | | | Time of day | |
| --- | --- | --- | --- | --- | --- |
|  | n | *r* | *p* | *r* | *p* |
| *Andropadus virens* |  |  |  |  |  |
| Residual mass | 105 | -0.064 | 0.519 | -0.064 | 0.517 |
| Hct (%) | 93 | 0.095 | 0.366 | -0.042 | 0.690 |
| Weight (g) | 105 | -0.063 | 0.523 | -0.065 | 0.508 |
| Hb (g/L) | 93 | 0.043 | 0.683 | -0.084 | 0.421 |
| Lymph (%) | 93 | 0.018 | 0.866 | 0.035 | 0.739 |
| Heterophils (%) | 93 | 0.097 | 0.353 | 0.056 | 0.594 |
| H/L ratio | 93 | 0.104 | 0.322 | 0.067 | 0.524 |
| Subcutaneous Fat | 105 | 0.074 | 0.450 | 0.022 | 0.825 |
| Muscle score | 105 | 0.060 | 0.540 | 0.046 | 0.639 |
| *Andropadus latirostris* | |  |  |  |  |
| Residual mass | 126 | -0.068 | 0.452 | 0.159 | 0.076 |
| Hct (%) | 114 | 0.235 | 0.235 | 0.121 | 0.199 |
| Weight (g) | 126 | 0.067 | 0.459 | 0.143 | 0.110 |
| Hb (g/L) | 114 | 0.211 | 0.024 | 0.108 | 0.252 |
| Lymphocytes (%) | 114 | 0.068 | 0.474 | 0.026 | 0.783 |
| Heterophils (%) | 114 | 0.170 | 0.070 | -0.146 | 0.121 |
| H/L ratio | 114 | 0.161 | 0.086 | -0.141 | 0.133 |
| Subcutaneous Fat | 126 | -0.196 | 0.058 | -0.054 | 0.548 |
| Muscle score | 126 | 0.016 | 0.860 | 0.052 | 0.566 |

| Condition indices | | | Julian date | | Time since sunrise | |
| --- | --- | --- | --- | --- | --- | --- |
|  | | *n* | *r* | *p* | *r* | *p* |
| *Alethe diademata* | |  |  |  |  |  |
| Residual mass | | 59 | -0.098 | 0.461 | 0.000 | 0.997 |
| Hct (%) | | 54 | 0.262 | 0.058 | -0.125 | 0.371 |
| Weight (g) | | 59 | -0.073 | 0.582 | 0.039 | 0.769 |
| Hb (g/L) | | 54 | -0.105 | 0.456 | 0.168 | 0.230 |
| Lymphocytes (%) | | 54 | -0.105 | 0.456 | -0.040 | 0.777 |
| Heterophils (%) | | 54 | -0.371 | 0.060 | 0.168 | 0.230 |
| H/L ratio | | 54 | -0.248 | 0.073 | 0.152 | 0.276 |
| Subcutaneous Fat | | 59 | 0.133 | 0.316 | -0.010 | 0.938 |
| Muscle score | | 59 | 0.110 | 0.409 | 0.062 | 0.641 |
| *Cyanomitra obscura* | | |  |  |  |  |
| Residual mass | 116 | | 0.023 | 0.804 | -0.022 | 0.811 |
| Hct (%) | 101 | | -0.058 | 0.561 | -0.020 | 0.844 |
| Weight (g) | 116 | | 0.049 | 0.605 | -0.003 | 0.972 |
| Hb (g/L) | 101 | | 0.159 | 0.112 | -0.077 | 0.441 |
| Lymphocytes (%) | 101 | | 0.121 | 0.227 | -0.080 | 0.426 |
| Heterophils (%) | 101 | | -0.167 | 0.095 | -0.047 | 0.641 |
| H/L ratio | 101 | | -0.223 | 0.025 | -0.037 | 0.717 |
| Subcutaneous Fat | 116 | | -0.010 | 0.913 | -0.053 | 0.573 |
| Muscle score | 116 | | -0.103 | 0.273 | -0.136 | 0.145 |

**S3 Table** (continued)
