# Supplementary material for: Matrix Intensification Affects Body and Physiological Condition of Tropical Forest-Dependent Passerines
Source: PLoS One. 2015 Jun 24;10(6):e0128521. doi: 10.1371/journal.pone.0128521 (PMC4479600; doi:10.1371/journal.pone.0128521)
Supplement: S4 Table — (DOCX) [file pone.0128521.s005.docx]

**S4 Table:** Model-averaged coefficients from models of residual body mass, H/L ratio and fat score for each target species and sum of Akaike weights (Σωi) for each explanatory variable.

| Response | Variable | Coefficient | SE | LCI | UCI | Σωi |
| --- | --- | --- | --- | --- | --- | --- |
| *Andropadus virens* |  |  |  |  |  |  |
| Residual mass | Dist.edge*Matrix | 0.96 | 0.41 | 0.15 | 1.76 | 0.09 |
|  | Large trees | -0.09 | 0.23 | -0.54 | 0.36 | 0.10 |
|  | Forest extent | 0.04 | 0.22 | -0.39 | 0.47 | 0.10 |
|  | Matrix[Mining] | 0.10 | 0.13 | -0.16 | 0.35 | 0.14 |
|  | Dist.edge [Interior] | 0.00 | 0.46 | -0.91 | 0.91 | 0.25 |
|  | Fruiting trees | 0.01 | 0.02 | -0.02 | 0.04 | 0.38 |
| H/L ratio | Dist.edge*Matrix | 0.19 | 0.15 | -0.10 | 0.47 | 0.00 |
|  | Fruiting trees | 0.00 | 0.00 | -0.01 | 0.00 | 0.00 |
|  | Large trees | 0.02 | 0.02 | -0.03 | 0.07 | 0.20 |
|  | Forest extent | 0.01 | 0.03 | -0.05 | 0.06 | 0.20 |
|  | Dist.edge [Interior] | 0.02 | 0.07 | -0.13 | 0.17 | 0.40 |
|  | Matrix [Mining] | -0.05 | 0.04 | -0.13 | 0.03 | 0.40 |
| Subcutaneous fat | Fruiting trees | 0.00 | 0.00 | 0.00 | 0.01 | 0.00 |
|  | Dist.edge*Matrix | 0.10 | 0.15 | -0.20 | 0.41 | 0.02 |
|  | Large trees | -0.04 | 0.04 | -0.11 | 0.03 | 0.03 |
|  | Forest extent | 0.08 | 0.04 | 0.01 | 0.16 | 0.14 |
|  | Matrix [Mining] | -0.17 | 0.08 | -0.32 | -0.01 | 0.30 |
|  | Dist.edge [Interior] | 0.25 | 0.08 | 0.09 | 0.41 | 0.75 |

**S4 Table** (continued)

| Response | Variable | Coefficient | SE | LCI | UCI | Σωi |
| --- | --- | --- | --- | --- | --- | --- |
| *Andropadus latirostris* | |  |  |  |  |  |
| Residual mass | Forest extent | 0.56 | 0.16 | 0.24 | 0.88 | 0.09 |
|  | Large trees | 0.21 | 0.17 | -0.12 | 0.53 | 0.12 |
|  | Fruiting trees | 0.25 | 0.17 | -0.09 | 0.58 | 0.13 |
|  | Dist.edge*Matrix | 0.35 | 0.72 | -1.07 | 1.77 | 0.16 |
|  | Dist.edge [Interior] | 0.38 | 0.41 | -0.41 | 1.18 | 0.16 |
|  | Matrix [Mining] | -0.69 | 0.35 | -1.37 | 0.00 | 0.58 |
| H/L ratio | Dist.edge*Matrix | 0.00 | 0.04 | -0.08 | 0.08 | 0.02 |
|  | Fruiting trees | 0.01 | 0.02 | -0.02 | 0.04 | 0.02 |
|  | Large trees | 0.01 | 0.02 | -0.02 | 0.05 | 0.02 |
|  | Forest extent | 0.02 | 0.02 | -0.01 | 0.06 | 0.03 |
|  | Distance[Interior] | -0.03 | 0.03 | -0.10 | 0.03 | 0.05 |
|  | Matrix[Mining] | -0.05 | 0.03 | -0.11 | 0.01 | 0.08 |
| Subcutaneous fat | Forest extent | 0.01 | 0.23 | -0.45 | 0.46 | 0.15 |
|  | Fruiting trees | 0.19 | 0.20 | -0.21 | 0.58 | 0.18 |
|  | Dist.edge*Matrix | 0.04 | 0.69 | -1.31 | 1.39 | 0.25 |
|  | Large trees | 0.27 | 0.22 | -0.17 | 0.71 | 0.29 |
|  | Dist.edge[Interior] | 1.12 | 0.41 | 0.31 | 1.93 | 0.75 |
|  | **Matrix[Mining]** | **-0.62** | **0.43** | **-1.47** | **0.23** | **0.99** |

| Response Variable | | Coefficient | SE | LCI | UCI | Σωi |
| --- | --- | --- | --- | --- | --- | --- |
| *Alethe diademata* |  |  |  |  |  |  |
| Residual mass | Large trees | 0.00 | 0.14 | -0.28 | 0.29 | 0.02 |
|  | Dist.edge*Matrix | 0.58 | 0.70 | -0.80 | 1.96 | 0.12 |
|  | Forest extent | 0.17 | 0.20 | -0.23 | 0.57 | 0.15 |
|  | Fruiting trees | -0.34 | 0.15 | -0.64 | -0.05 | 0.21 |
|  | Dist.edge [Interior] | -0.53 | 0.57 | -1.64 | 0.59 | 0.35 |
|  | **Matrix[Mining]** | **-3.10** | **0.72** | **-4.50** | **-1.69** | **1.00** |
| H/L ratio | Dist.edge*Matrix | -0.05 | 0.13 | -0.31 | 0.21 | 0.01 |
|  | Large trees | -0.01 | 0.00 | -0.01 | 0.00 | 0.01 |
|  | Fruiting trees | 0.02 | 0.03 | -0.04 | 0.07 | 0.03 |
|  | Forest extent | -0.02 | 0.05 | -0.11 | 0.07 | 0.04 |
|  | Dist.edge [Interior] | -0.03 | 0.07 | -0.16 | 0.10 | 0.07 |
|  | **Matrix [Mining]** | **0.28** | **0.07** | **0.14** | **0.41** | **0.96** |
| Subcutaneous fat | Large trees | -0.06 | 0.03 | -0.12 | 0.00 | 0.03 |
|  | Fruiting trees | 0.07 | 0.22 | -0.37 | 0.51 | 0.10 |
|  | Dist.edge*Matrix | -1.15 | 0.73 | -2.59 | 0.29 | 0.14 |
|  | Forest extent | 0.33 | 0.27 | -0.20 | 0.86 | 0.22 |
|  | Matrix [Mining] | 0.72 | 0.64 | -0.54 | 1.98 | 0.37 |
|  | Dist.edge [Interior] | 0.93 | 0.50 | -0.06 | 1.91 | 0.64 |

**S4** **Table** (continued)

| Response Variable | | Coefficient | SE | LCI | UCI | Σωi |
| --- | --- | --- | --- | --- | --- | --- |
| *Cyanomitra obscura* |  |  |  |  |  |  |
| Residual mass | Dist.edge*Matrix | 0.15 | 0.70 | -2.12 | 0.63 | 0.09 |
|  | Fruiting trees | -0.09 | 0.23 | -0.54 | 0.36 | 0.10 |
|  | Large trees | 0.04 | 0.22 | -0.39 | 0.47 | 0.10 |
|  | Forest extent | 0.00 | 0.46 | -0.91 | 0.91 | 0.25 |
|  | Dist.edge [Interior] | 0.01 | 0.02 | -0.02 | 0.04 | 0.38 |
|  | **Matrix[Mining]** | **-1.05** | **0.43** | **-1.90** | **-0.21** | **0.95** |
| H/L ratio | Dist.edge*Matrix | -0.06 | 0.05 | -0.16 | 0.05 | 0.00 |
|  | Fruiting trees | 0.00 | 0.00 | 0.00 | 0.00 | 0.00 |
|  | Large trees | 0.01 | 0.02 | -0.03 | 0.05 | 0.02 |
|  | Forest extent | 0.01 | 0.02 | -0.03 | 0.04 | 0.02 |
|  | Dist.edge [Interior] | 0.03 | 0.03 | -0.03 | 0.08 | 0.04 |
|  | Matrix [Mining] | -0.10 | 0.05 | 0.00 | 0.20 | 0.21 |
| Subcutaneous fat | Fruiting trees | 0.04 | 0.02 | 0.00 | 0.08 | 0.01 |
|  | Large trees | 0.17 | 0.22 | -0.27 | 0.61 | 0.20 |
|  | Dist.edge*Matrix | 0.99 | 0.63 | -0.24 | 2.22 | 0.32 |
|  | Forest extent | 0.27 | 0.18 | -0.08 | 0.62 | 0.33 |
|  | Dist.edge [Interior] | 0.28 | 0.48 | -0.66 | 1.23 | 0.65 |
|  | Matrix [Mining] | -1.00 | 0.70 | -2.37 | 0.36 | 0.75 |

**S4 Table** (continued)
